# Tipps und Tricks für die Zemententfernung im Revisionsfall

**DOI:** 10.1007/s00132-023-04453-8

**Published:** 2023-10-19

**Authors:** Marc-Pascal Meier, Thelonius Hawellek, Wolfgang Lehmann, Gabriela von Lewinski

**Affiliations:** Klinik für Unfallchirurgie, Orthopädie und Plastische Chirurgie, Universitätsmedizin Göttingen, Robert-Koch-Str. 40, 37075 Göttingen, Deutschland

**Keywords:** Endoprothetik, Knochenzement, Chirurgische Revision, Totaler Hüftgelenkersatz, Totaler Kniegelenkersatz, Arthroplasty, Bone cement, Surgical revision, Total hip replacement, Total knee replacement

## Abstract

**Hintergrund:**

Aktuelle Re-Revisionsraten nach endoprothetischem Gelenkersatz liegen in Deutschland bei 28–37 %. Insbesondere verbliebende Zementreste sind für erneute Revisionseingriffen nach Sanierungsoperationen bei periprothetischen Infektionen ursächlich, weswegen die vollständige Zemententfernung von großer Bedeutung ist. Die Entfernung letzter Zementreste stellt den Operateur jedoch häufig vor technische Herausforderungen. Eine komplikationslose und vollständige Zemententfernung bedarf einer umfangreichen präoperativen Vorbereitung, um die bestmögliche Operationsstrategie zu entwickeln.

**Therapie:**

Von verschiedenen Herstellern werden Spezialinstrumente angeboten, die die Zemententfernung im Revisionsfall erleichtern. Neben endoluminalen Zugängen existieren Zugangserweiterungen wie zusätzliche Osteotomien, welche die vollständige Zemententfernung erleichtern. Nicht zuletzt sollte der Operateur in der Lage sein, nach einem definierten Zeitintervall die Indikation zum intraoperativen Vorgehenswechsel zu stellen.

## Abstract (en)

**Background:**

In Germany, current revision rates after arthroplasty range between 28–37%. In particular, remaining cement residues are causative for additional revision surgery after periprosthetic joint infection, which is why complete cement removal is of high importance. However, the removal of remaining cement residues often confronts the surgeon with technical challenges. Complication-free and complete cement removal requires extensive preoperative preparation in order to develop the best possible surgical strategy.

**Treatment:**

Special instrument sets to facilitate cement removal in revision cases are available from various manufacturers. In addition to endoluminal approaches, access enhancements such as extended osteotomies exist to facilitate complete cement removal. Finally, the surgeon should be able to give the indication for an intraoperative procedural change after a defined time interval.

In der Revisionsendoprothetik ist die gewissenhafte und vollständige Zemententfernung von großer Bedeutung. Intraoperativ stellt jedoch die Entfernung letzter Zementreste den Operateur häufig vor technische Herausforderungen. Der nachfolgende Beitrag soll Tipps und Tricks für eine erfolgreiche und komplikationslose Zemententfernung bei Wechseloperationen geben.

## Einleitung

Im Jahr 2021 wurden in Deutschland 158.690 primär implantierte Hüftgelenkendoprothesen und 115.581 primäre Kniegelenkendoprothesen im Endoprothesenregister Deutschland (EPRD) erfasst [1]. Nach den Registerdaten wurden 17.752 Revisionseingriffe am Hüftgelenk und 13.961 am Kniegelenk durchgeführt [1]. Unter anderem aufgrund des demografischen Wandels wird bis zum Jahr 2030 ein Anstieg der Revisionseingriffe um ca. 34 % erwartet [2–4]. Die aktuellen Re-Revisionsraten liegen umfassend bei 28–37 % [5]. Vor allem bei septischen Wechseleingriffen sind verbliebende Zementreste relevant ursächlich für neuerlich notwendige Revisionsoperationen. Darüber hinaus können verbliebende Zementreste auch mechanische Komplikationen bedingen [6, 7]. Daher kommt der gewissenhaften und vollständigen Zemententfernung im Revisionsfall eine große Bedeutung zu. Diese stellt den Operateur jedoch häufig vor technische Herausforderungen. Der vorliegende Beitrag soll Tipps und Tricks aufzeigen, um die Herausforderungen erfolgreich zu bewältigen.

## Präoperative Planung

Revisionsoperationen bedürfen einer peniblen präoperativen Vorbereitung. Hier gilt es, möglichst viele Informationen über den Patienten und die einliegenden Implantate einzuholen. Um die bestmögliche Strategie für den Wechseleingriff zu entwickeln, sollten neben der grundlegenden krankheitsspezifischen Anamnese folgende Punkte geklärt werden:

- Welche Indikation begründet die Revisionsoperation (aseptische Lockerung, periprothetische Infektion, periprothetische Fraktur)?
- Welche Implantate/Fremdmaterialien liegen aktuell in situ ein?
- Welcher Zugang/welche Zugänge wurden in Voroperationen genutzt?
- Welche Knochenqualität weist der Patient auf bzw. bestehen ossäre Defektsituationen?
- Welche Instrumente werden für die Prothesen- und Zementexplantation benötigt?

Diese Fragen sind essenziell für die präoperative Planung der Zemententfernung im Revisionsfall und sollen nachfolgend vertieft werden.

### Revisionsindikation

Die Revisionsindikation nimmt Einfluss auf das Ausmaß der Zemententfernung [8–10]. Im Falle einer periprothetischen Infektion wird die radikale und vollständige Zemententfernung von den Autoren empfohlen. Bei aseptischen Prothesenlockerungen werden in der aktuellen Literatur zwei Strategien diskutiert [6, 10, 11]. Eine Möglichkeit besteht im Wechsel einer zementierten Prothese auf eine zementfreie Variante [12, 13]. Demgegenüber steht die Zement-in-Zement-Revisionstechnik [14, 15]. Entscheidet sich der Operateur in der Revision für einen Wechsel des Verankerungstyps, wird von den Autoren ebenfalls die vollständige Zemententfernung empfohlen.

Je nach Frakturmorphologie kann eine Zementerhaltung oder -entfernung diskutiert werden

Je nach Frakturmorphologie kann eine Zementerhaltung oder -entfernung diskutiert werden. Undislozierte periprothetische Frakturen ohne Anhaltspunkte für eine Implantatlockerung ermöglichen in der Regel einen Erhalt des Zementmantels. Dislozierte Frakturen, welche mit einer Implantatlockerung einhergehen, indizieren häufig eine vollständige Zemententfernung [16, 17].

### Implantate und Fremdmaterial

Essenziell für eine adäquate präoperative Planung ist die Kenntnis über die einliegenden Implantate und ggf. weitere Fremdmaterialien. Zu diesem Zweck sollte vor jedem Revisionseingriff der Implantatausweis angefordert werden. Präoperativ muss geklärt werden, ob das passende Explantationsinstrumentarium in domo vorhanden ist oder bestellt werden muss. Zur komplikationsarmen Entfernung, insbesondere bei noch fest integrierten Prothesenanteilen, empfehlen die Autoren die Teilkomponentenexplantation oder vollständige Explantation sowie Zemententfernung dezidiert präoperativ zu planen [18].

### Zugang

Die Kenntnis über den verwendeten Zugangsweg bei Voroperationen ist von enormer Bedeutung. In Abhängigkeit von diesem muss der Operateur entscheiden, ob eine Zugangserweiterung für den Revisionseingriff notwendig ist. Eventuell muss aus Gründen der Übersicht bzw. der Exploration ein zusätzlicher Zugang angelegt werden [19, 20]. Bereits präoperativ sollte eine Strategie bestehen, die eine vollständige Zemententfernung ermöglicht.

### Knochenqualität und ossäre Defekte

Eine verminderte Knochenqualität und ossäre Defekte erhöhen das Komplikationsrisiko bei der Zemententfernung im Revisionsfall. Die häufigste Major-Komplikation ist die iatrogene Fraktur [21–23]. Präoperativ sollen frakturgefährdete Areale anhand der durchgeführten Bildgebung identifiziert werden und in die Planung des intraoperativen Vorgehens einbezogen werden. Insbesondere im Bereich von Defektstellen ist ein bedachtes Vorgehen bei der Zemententfernung notwendig [24, 25].

### Auswahl der Instrumente

Je nachdem welche Revisionsinstrumente in der eigenen Klinik vorhanden sind, empfiehlt es sich in manchen Fällen Spezialinstrumente anzufordern. Insbesondere bei bereits mehrfach voroperierten Patienten bestehen nicht selten langstreckige Zementierungen. Die vollständige Entfernung des gesamten Zementmantels ist häufig mit den Standardinstrumenten stark erschwert bis nicht möglich [18]. Erschwerte Bedingungen steigern das Risiko für Komplikationen. Daher sollte in der präoperativen Planung besonderes Augenmerk auf der Auswahl der Revisionsinstrumente liegen [26, 27].

#### *Tipp 1:*

Holen Sie möglichst viele Informationen über den Patienten und seine Implantate ein, um die bestmögliche Strategie für die Zemententfernung zu entwickeln.

## Präoperative Diagnostik

Ein Standard in der präoperativen Diagnostik vor Revisionseingriffen ist die Röntgenbildgebung in zwei Ebenen. Hier muss darauf geachtet werden, dass die einliegenden Implantate sowie der Zement ausreichend abgebildet sind. Die angrenzenden Gelenke sollten immer miterfasst sein. Aktuelle Studien belegen, dass eine konventionelle Bildgebung vor Revisionseingriffen nicht ausreicht, um komplexe Situationen vollständig zu erfassen [28–32]. Vor jedem endoprothetischen Revisionseingriff, bei dem eine vollständige Zemententfernung geplant ist, wird präoperativ die Durchführung einer CT empfohlen. Durch diese kann die exakte Konfiguration des Zementmantels erfasst werden. Des Weiteren ist eine bessere Beurteilung knöcherner Defektsituationen möglich. Darüber hinaus gibt die CT-Bildgebung Rückschluss auf Integration und Festigkeit der einliegenden Implantate. Zusammenfassend lässt sich durch die CT der präoperative Prothesen- und Zementstatus besser erfassen [29]. Der Mehrgewinn von Informationen hilft bei der Festlegung der richtigen Operationsstrategie und verringert das Komplikationsrisiko.

### *Tipp 2:*

Veranlassen Sie vor dem Revisionseingriff immer eine CT-Diagnostik, um den einliegenden Zementmantel exakt erfassen zu können und knöcherne Schwachstellen zu identifizieren.

Tab. 1 zeigt eine Checkliste zur optimalen präoperativen Vorbereitung eines Patienten für einen Revisionseingriff.

**Table 1:** 

Präoperative Checkliste	
Implantatausweis vorhanden?	□
Wechselimplantate vorhanden? Wenn nein, sind diese bestellt?	□
Bei hausfremden Systemen: Leihsiebe bestellt?	□
Revisionsinstrumente vorhanden? Wenn nein, sind diese bestellt?	□
Röntgen- und CT-Bildgebung erfolgt?	□
Erhöhtes Frakturrisiko?	□
Ggf. notwendige Osteosynthesematerialien vorhanden?	□
Operationsstrategie festgelegt?	□

## Instrumentarien zur Zemententfernung

Bei der Zemententfernung im Revisionsfall ist die Schonung der Weichteile und des Knochens von großer Bedeutung. Daher empfiehlt es sich, auf spezielle Explantationsinstrumente zurückzugreifen [18, 27, 33]:

- Meißel: Eine großzügige Auswahl an Meißeln sollte der Standard für Revisionsoperationen sein, bei den der eingebrachte Knochenzement vollständig entfernt werden soll. Neben starren Instrumenten können flexible und pneumatische Meißel die Zementexplantation erleichtern. Speziell konfektioniert für die Zemententfernung aus dem Azetabulum (Pfannenrandmeißel) und dem Markraum langer Röhrenknochen, werden Meißel in verschiedenen Varianten von unterschiedlichen Herstellern angeboten. Diese begünstigen eine knochensparende Zemententfernung und sind deshalb zu empfehlen.
- Fräsen: Bei festintegrierten Prothesenschäften und intaktem Zementinterface kann die Explantation deutlich erschwert sein. Um eine möglichst knochensparende Entfernung durchzuführen, eignen sich Hochfrequenzfräsen. Hohlfräsen ermöglichen ein Überbrücken von abgebrochenen „Zementstielen“, sodass diese sekundär geborgen werden können.
- Bohrer: Ein breites Sortiment an verschiedenen Bohrern kann die Zemententfernung im Revisionsfall deutlich erleichtern. Neben Bohrern mit Zentrierhülsen und nicht schneidenden Spitzen, erweisen sich auch kanülierte Bohrer als hilfreich. In Seldinger-Technik können so tief intramedullär einliegende Zementreste vorbereitet werden, um schließlich mit langen Greifinstrumenten geborgen zu werden [27].
- Rongeur: Rongeure sind bestens geeignet, um bereits losgelöste tief intramedullär gelegene Zementreste zu fassen und sicher zu entfernen. Sofern greifbar, ist auch eine Entfernung noch einliegender Zementstopper möglich.
- Osteotom/Spalter: Zur Vermeidung ungewollter intraoperativer Frakturen sowie zur leichteren Zemententfernung können Osteotomien zweckdienlich sein. Um eine möglichst komplikationsarme Durchführung zu gewährleisten, können verschiedenen Osteotome/Spalter und Osteotom-Wedges genutzt werden.

**Figure 1:**
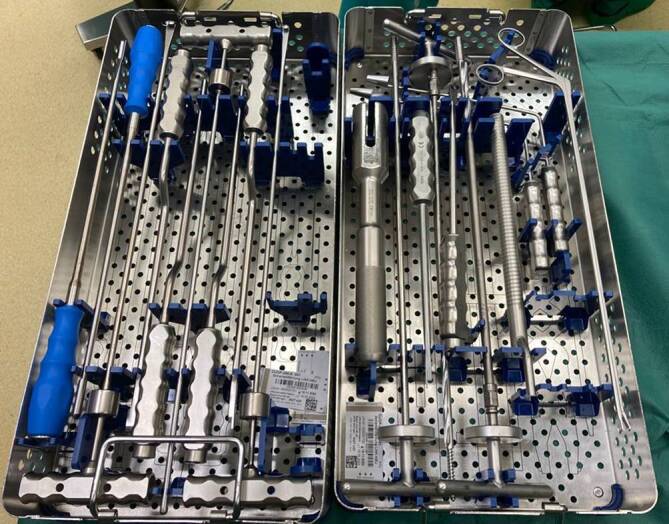

Abb. 1 zeigt eine Auswahl an Standardinstrumenten für die Zemententfernung im Revisionsfall.

### *Tipp 3:*

Die Auswahl der richtigen Explantationsinstrumente ermöglicht eine weichteil- und knochensparende Zemententfernung.

## Operative Strategien

Die Explantation zementierter Endoprothesen mit dem Ziel eines möglichst weichteil- und knochensparenden Ausbaus ist anspruchsvoll. Standardimplantate lassen sich häufig durch endofemorale oder endotibiale Zugänge komplikationslos entfernen. Bei Revisionsimplantaten bzw. guter Integration des Zementinterfaces kann die Explantation jedoch erschwert sein. Auch das Bergen von tiefgelegenen Zementresten und von Zementstoppern stellt öfters eine Herausforderung dar. In diesen Fällen empfehlen die Autoren eine Zugangserweiterung. Nachfolgend werden ausgewählte operative Strategien zur Zemententfernung im Revisionsfall an Hüft- und Kniegelenk näher erläutert.

### Endofemoraler und endotibialer Zugang

Gelockerte Implantate können nach Freilegung der gelenknahen Komponentenanteile häufig durch geeignete Ausschlaginstrumente explantiert werden. Bei fest integriertem Zementmantel können schlanke gerade Meißel verwendet werden, um die Verbindung zwischen Zement und Knochen vorsichtig aufzulösen. Hier besteht jedoch das Risiko einer iatrogenen Fraktur. Insbesondere sollte darauf geachtet werden, keine Perforation zu verursachen. Mittels Rongeur können bereits gelöste Zementreste aus tiefer gelegenen Markraumteilen entfernt werden. Darüber hinaus besteht die Möglichkeit einer Markraumaufbohrung. Um Frakturen zu vermeiden, muss hier eine mögliche Ausdünnung der Kortikalis kritisch evaluiert werden. Kontinuierliche Durchleuchtungskontrollen sind bei dünner Kortikalis zwingend erforderlich. Zur Entfernung des Zementstoppers können Korkenzieherküretten genutzt werden. Auch eine Überbohrung ist möglich [20, 27, 34, 35].

Bei zu hohem Frakturrisiko oder schlechter Exposition über den endoluminalen Zugang ist die Indikation zur Osteotomie großzügig zu stellen [36].

#### *Trick 1:*

Besteht ein zu hohes Frakturrisiko oder reicht die Exposition über einen endoluminalen Zugang nicht aus, wird die Durchführung einer zusätzlichen Osteotomie empfohlen.

### Transfemoraler Zugang

#### Wagner-Osteotomie

**Figure 2:**
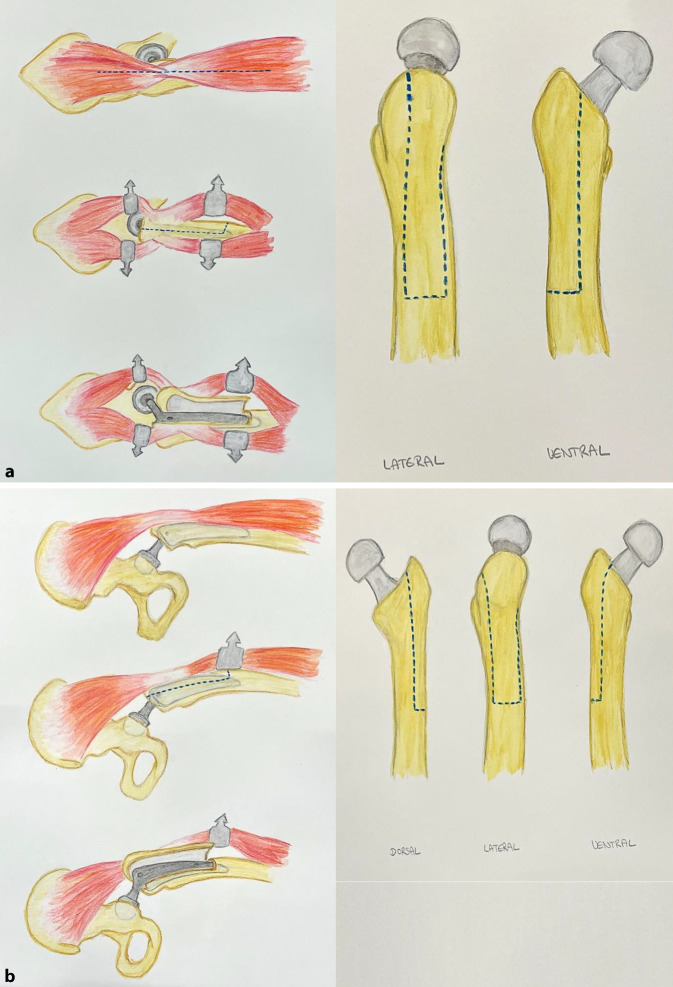

Im Falle eines verwendeten anterolateralen/lateralen Zugangs zum Hüftgelenk kann zur Schaftexplantation oder zur Entfernung von Zementresten eine Wagner-Osteotomie erfolgen. Hierzu ist zunächst die Präparation der vastuglutealen Schlinge notwendig. Anschließend wird der Vastus lateralis gespalten. Nach Freilegung des proximalen Femurs empfiehl sich die Anlage von Bohrlöchern zur Begrenzung der folgenden Osteotomie. Ein Bohrer der Stärke 3,2 mm eignet sich hierzu sehr gut. Mittels schmalem Sägeblatt wird das Femur lateralseitig von proximal nach distal osteotomiert. Der Knochendeckel kann nun nach ventral abgehoben werden (Abb. 2). Zum Schutz vor iatrogenen Frakturen wird empfohlen, die Breite des Knochendeckels kleiner als ein Drittel der Femurdiaphyse zu wählen. Nach erfolgter Schaftexplantation und Zemententfernung sollte der Knochendeckel mittels Cerclagen refixiert werden [27, 37, 38].

#### Erweiterte Trochanterosteotomie

Bei Revisionen über den dorsalen Zugang zum Hüftgelenk erfolgt die Anlage der Trochanterosteotomie von dorsolateral. Als Leitstruktur dient die Linea aspera. Diese wird über einen Subvastuszugang freigelegt. Die Begrenzung des Knochendeckels wird ebenfalls durch Bohrlöcher markiert. Alternativ können die Grenzen mit einem feinen Meißel gesetzt werden. Die Länge des Knochendeckels richtet sich nach Länge des einliegenden Prothesenschaftes bzw. der Tiefe des Zementstoppers. In der Regel liegt diese zwischen 12–15 cm [27, 39, 40]. Nach Vollendung der Sägeschnitte kann der Knochendeckel mittels Osteotom nach lateral abgehoben werden (Abb. 2). Wie zuvor beschrieben, erfolgt dessen Refixation nach vollständiger Explantation mittels Cerclagen.

#### Femorales Knochenfenster

**Figure 3:**
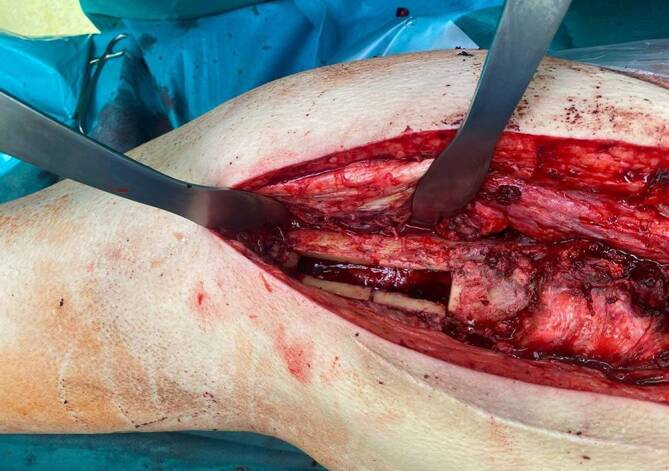

Bei implantierten Langschäften oder tief diaphysär gelegenen Zementresten/-stoppern ist die Anlage eines Knochenfensters am distalen Femur eine gute Option für eine vollständige Entfernung letzter Fremdmaterialien und Zementreste. Für eine ausreichend gute Übersicht empfehlen die Autoren auch bei diesem Vorgehen das Ablösen der vastuglutealen Schlinge. Vor Zurichtung des Knochenfensters sollten dessen Begrenzung – wie zuvor beschrieben – mittels Bohrlöcher oder feinem Meißel markiert werden. Nach Zurichtung kann der Knochendeckel vollständig abgehoben werden (Abb. 3). Hier ist darauf zu achten, das Knochenfenster in ausreichender Größe anzulegen, um genügend Platz zum Einführen der Explantationsinstrumente zu schaffen. Unter Berücksichtigung biomechanischer Gesichtspunkte ist die Anlage eines trapezförmigen Knochenfenster zu empfehlen. Diese und gewinkelte Osteotomien erleichtern die spätere Refixation und bieten eine höhere Stabilität [27]. Dem gegenüber haben rechteckige Osteotomien den Vorteil, dass eine geringe Gesamtlänge des Knochenfenster benötigt wird, da der Bewegungsspielraum für die eingebrachten Instrumente bei gleicher Länge höher ausfällt. [27, 39]. Nach vollständiger Entfernung der Zementreste und Fremdmaterialien wird der Knochendeckel mittels Cerclagen refixiert [41–43].

### Erweiterte Zugänge in der Kniegelenksrevision

#### Tuberositas-tibiae-Osteotomie (TTO)

**Figure 4:**
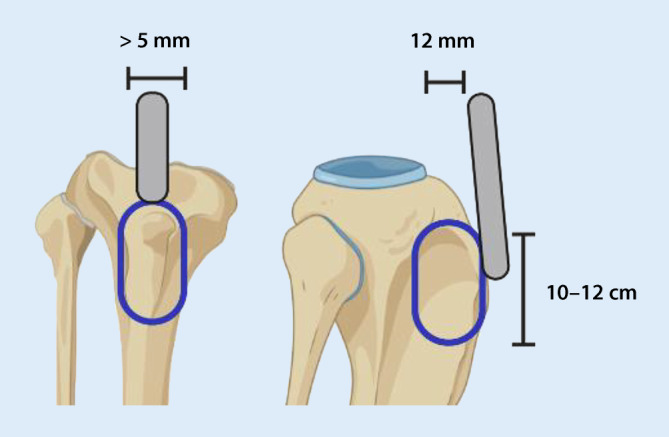

In der Knierevisionsendoprothetik ist die TTO ein bewährtes Verfahren. Bei starkem Flexionsdefizit kann unter anderem eine unkontrollierte Schädigung der Patellarsehne verhindert werden. Darüber hinaus ist die TTO eine Option, wenn das herkömmliche laterale Release nicht ausreichend Übersicht verschafft. Die TTO kann sowohl bei Voroperationen über einen medialen als auch lateralen Zugang verwendet werden. Außerdem besteht die Möglichkeit einer Korrektur von patellaren Fehlstellungen. Die Autoren empfehlen eine Markierung der Osteotomiekanten durch Bohrlöcher. Als Minimum für die Osteotomiedicke werden 5 mm empfohlen, für die Osteotomiebreite 12 mm. Zur Vermeidung von Stressfrakturen sollte die Osteotomie nach dorsal gebogen und nach distal auslaufend angelegt werden (Abb. 4). Die bevorzugte Methode zur Refixation ist die Schraubenosteosynthese mittels zwei bis drei 4,5-mm-Kortikalisschrauben [34, 44–47].

#### „Rectus snip“ nach Insall

**Figure 5:**
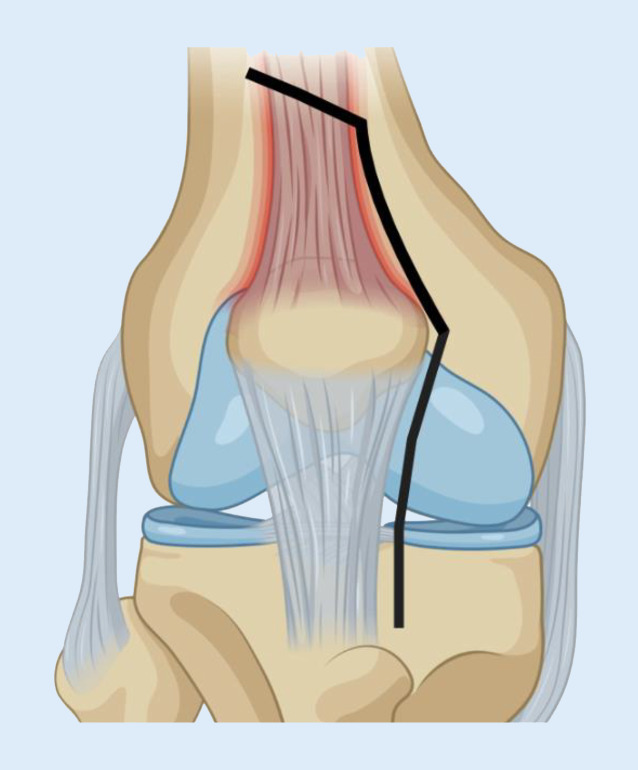

Ist trotz umfangreichem Release aufgrund einer zu hohen Spannung der Quadrizepssehne keine Eversion der Patella möglich, eignet sich der „rectus snip“ nach Insall als Zugangserweiterung. Hierbei wird die Quadrizepssehne im Vorlauf der Arthrotomie nach proximal im 45°-Winkel nach lateral inzidiert. Sollte dies nicht ausreichen, um die Patella spannungsfrei zu lateralisieren, kann die Inzision 1–2 cm im Faserverlauf des M. vastus lateralis fortgesetzt werden (Abb. 5). Diese Zugangserweiterung bietet die Möglichkeit eines einfachen Wundverschlusses, weist gute Heilungstendenzen auf und schont die lateralen Gefäße [45, 48]. Der Inzisionsdefekt muss im Rahmen des Wundverschlusses gewissenhaft refixiert werden.

#### Tibiales Knochenfenster

Analog zum oben beschriebenen Vorgehen am distalen Femur ist auch die Anlage eines Knochenfensters an der distalen Tibia möglich. Lange Stems können leichter als mit der endotibialen Technik entfernt werden. Wie zuvor beschrieben, erleichtert die Anlage eines tibialen Knochenfensters auch die Entfernung einliegender Zementstopper. Zur Markierung des geplanten Knochenfensters wird ebenfalls eine Zurichtung mittels Meißel oder Bohrlöchern empfohlen. Je nach Größe des abgehobenen Knochendeckels kann teilweise von einer osteosynthetischen Refixation abgesehen werden [49, 50].

Tab. 2 zeigt eine Zusammenfassung möglicher Zugänge zur Zemententfernung in der Hüft- und Kniegelenkrevision.

**Table 2:** 

Hüftgelenkrevision	Kniegelenkrevision
Endofemoraler Zugang	Endotibialer Zugang
Wagner-Osteotomie	Tuberositas-tibiae-Osteotomie
Erweiterte Trochanterosteotomie	„Rectus snip“ nach Insall
Distales femorales Knochenfenster	Distales tibiales Knochenfenster

##### *Trick 2:*

Zugangserweiterungen bedeuten einen höheren operativen Aufwand, bieten bei korrekter Durchführung jedoch eine wesentlich bessere Implantat- und Zementexposition.

### Zemententfernung durch Ultraschall

Aktuelle Studien weisen darauf hin, dass die Zemententfernung durch Ultraschall zukünftig in Revisionsfällen in Erwägung gezogen werden sollte. Dieses Verfahren gilt allgemeinhin als knochenschonend und reduziert das Risiko für intraoperative Frakturen oder Perforationen. Aktuell fehlen jedoch ausreichend fundierte Daten zur Qualität dieser Methode. Darüber hinaus muss angemerkt werden, dass alle Vergleichsstudien zu diesem Thema von geringen, jedoch vorhandenen, Zementrückständen berichten. Dieser Umstand ist insbesondere in Bezug auf die Eradikationsrate bei periprothetischen Infektionen zu hinterfragen [51–54].

## Herausforderungen, Risiken und Komplikationen

Die Zemententfernung im Revisionsfall kann den Operateur vor gewisse Herausforderungen stellen. Nachfolgend sollen diese sowie intraoperative Risiken und Komplikationen genauer beleuchtet werden.

### Die schwierige Ausgangslage

**Figure 6:**
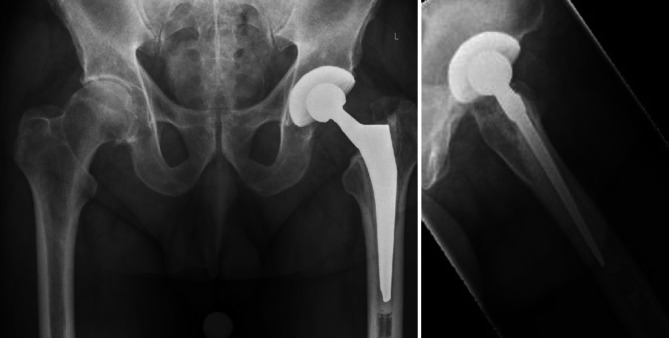

Eine Herausforderung für eine radikale Zemententfernung besteht bei asymmetrischem Zementmantel mit einer azentrischen Lage des Prothesenschaftes (Abb. 6). Aufgrund der nicht linear verlaufenden Einflugschneise ist die Zemententfernung über einen singulären endoluminalen Zugang deutlich erschwert, insbesondere wenn der proximale Schaftanteil unilateral an der Kortikalis anliegt und die Schaftspitze in unmittelbarer Nähe zur Gegenkortikalis zum Liegen kommt. In diesem Fall ist das Zementinterface häufig nur unilatreal zugänglich. Bei nur einseitiger Entfernung des Zementinterfaces und azentrischem Verlauf des Prothesenschaftes ist eine ausreichende Zementbefreiung zur Komponentenexplantation nicht gewährleistet. Des Weiteren steigt das intraoperative Frakturrisiko, da die Krafteinwirkung bei Zemententfernungsversuchen mit beispielsweise langen Meißeln nicht linear auf den Zementmantel einwirkt.

### Die iatrogene Fraktur

Komplexe Zemententfernungen weisen – wie bereits beschrieben – ein erhöhtes iatrogenes Frakturrisiko auf. Aus diesem Grund sollten Osteosynthesesiebe bereits in unmittelbarer Nähe zum Operationssaal vorbereitet werden. Je nach Frakturmorphologie eignen sich Cerclagen oder verschiedene Plattensysteme. Bei Frakturen im Bereich der Trochanterregion werden Krallenplatten zur Refixation empfohlen. Cerclagen sollten überwiegend im femoralen und tibialen Schaftbereich zu Anwendung kommen [55–57].

### Unvollständige Zemententfernung

Die unvollständige Zemententfernung stellt eine Komplikation bei der radikalen Sanierung einer periprothetischen Infektion durch vollständige Explantation dar [27, 54, 58]. Daher ist ein gewissenhaftes und selbstkritisches Vorgehen bei der Zemententfernung unerlässlich. Die Autoren empfehlen daher intraoperativ dynamische Durchleuchtungskontrollen durchzuführen. Außerdem kann die präoperativ durchgeführte CT-Untersuchung Hinweise auf möglicherweise in situ verbliebende Zementreste liefern und sollte intraoperativ zur Reverifizierung genutzt werden.

#### *Trick 3:*

Dynamische Durchleuchtungskontrollen sowie ein erneuter Abgleich mit der präoperativen CT erleichtern die Detektion von in situ verbliebenen Zementresten.

## Zeitmanagement – Cut off zum Strategiewechsel

Zeitmanagement spielt insbesondere bei älteren komorbiden Patienten eine wichtige Rolle. Mit zunehmender Operationszeit steigen der Blutverlust und das Letalitätsrisiko [59–61]. Daher sollte bereits präoperativ eine Cut-off-Zeit für einen Strategiewechsel bei frustraner Zemententfernung festgelegt werden. Bezogen auf die in diesem Beitrag vorgestellten operativen Strategien empfehlen die Autoren eine Cut-off-Zeit von einer Stunde zum Wechsel von endoluminalen Verfahren auf einen erweiterten Zugang wie oben beschrieben [17, 60].

### *Tipp 4:*

Nach einer Dauer von einer Operationsstunde wird bei frustraner Zemententfernung über einen endoluminalen Zugang der Wechsel auf einen erweiterten Zugang empfohlen.

## Fazit für die Praxis

- Für die Festlegung der bestmöglichen operativen Strategie werden umfangreiche Informationen über den Patienten benötigt.
- Eine CT-Bildgebung erleichtert die präoperative Revisionsplanung und die intraoperative Detektion von verbliebenden Zementresten.
- Der Einsatz von Spezialinstrumenten ermöglicht eine knochensparende Zemententfernung.
- Zugangserweiterungen sind aufwendiger, bieten jedoch eine bessere Exposition.
- Bei frustraner Zemententfernung über mehr als eine Stunde sollte ein Strategiewechsel erfolgen.

## Abkürzungen

EPRD: Endoprothesenregister Deutschland
TTO: Tuberositas-tibiae-Osteotomie
